# Diffusion ^19^F-NMR of Nanofluorides: In Situ Quantification of Colloidal Diameters and Protein Corona Formation in Solution

**DOI:** 10.1021/acs.nanolett.2c02994

**Published:** 2022-10-18

**Authors:** Reut Mashiach, Liat Avram, Amnon Bar-Shir

**Affiliations:** ^†^Department of Molecular Chemistry and Materials Science and ^‡^Department of Chemical Research Support, Weizmann Institute of Science, Rehovot, 7610001, Israel

**Keywords:** diffusion, ^19^F-NMR, nanocrystals, colloids, protein corona

## Abstract

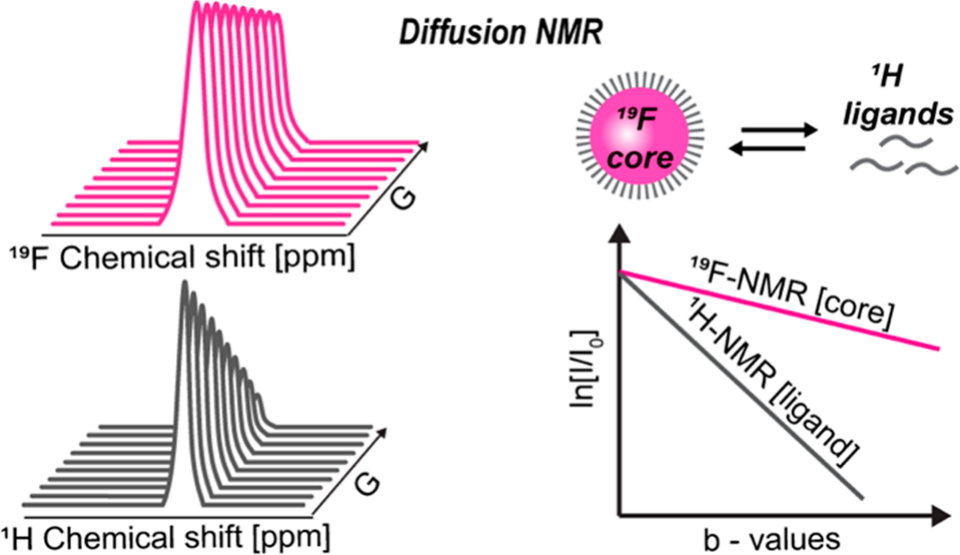

The NMR-detectability of elements of organic ligands that stabilize colloidal inorganic nanocrystals (NCs) allow the study of their diffusion characteristics in solutions. Nevertheless, these measurements are sensitive to dynamic ligand exchange and often lead to overestimation of diffusion coefficients of dispersed colloids. Here, we present an approach for the quantitative assessment of the diffusion properties of colloidal NCs based on the NMR signals of the elements of their inorganic cores. Benefiting from the robust ^19^F-NMR signals of the fluorides in the core of colloidal CaF_2_ and SrF_2_, we show the immunity of ^19^F-diffusion NMR to dynamic ligand exchange and, thus, the ability to quantify, with high accuracy, the colloidal diameters of different types of nanofluorides *in situ*. With the demonstrated ability to characterize the formation of protein corona at the surface of nanofluorides, we envision that this study can be extended to additional formulations and applications.

The diffusion properties of colloidal nanocrystals (NCs) in a complex medium are crucial in a wide range of applications.^1−6^ When obtained *in situ*, these properties do not only reflect the diameter of the colloids *de facto*, but also echo their actual mobility in their natural *milieu*. Among the analytical tools used to evaluate the diffusion coefficients of colloidal NCs, NMR studies are unique.^7−9^ Diffusion NMR measurements are applicable to opaque, cloudy, or colored solutions. They do not require sampling or dilution of the studied solution and there is no need for specific labeling of the colloids.^10,11^ Therefore, such measurements can be performed *in situ* to reflect the diffusion feature of the studied colloids in their intended environment.^12−27^ Nevertheless, diffusion NMR studies require NMR-detectable nuclei with appropriate relaxation properties and line-widths to obtain robust results. Thus, when applied to study dispersed inorganic NCs, they have, thus far, been limited to the elements of the organic surfactant ligands (^1^H, ^13^C, ^31^P, and even ^19^F) of the colloids. Relying on NMR signals of the ligands, these diffusion studies are frequently affected by dynamic ligand exchange and ligand excess, which often lead to overestimation of diffusion coefficients and essentially to underestimation of the evaluated colloidal diameters.^27−29^ This calls for further developments to make diffusion NMR studies independent of the organic–inorganic interface properties of dispersed colloidal inorganic NCs.

The NMR detectability of inorganic elements,^30^ which frequently reside at the core of many types of NCs,^10,31−36^ may provide an alternative solution. Nonetheless, the relatively low natural abundance of the NMR-detectable isotopes of these elements, their low gyromagnetic ratio, as well as their restricted mobility within the NC’s lattice, result in a very low NMR sensitivity.^37,38^ Therefore, their NMR signals are not applicable for diffusion measurements where the observation of NMR signal decays is mandatory. Colloidal nanofluorides (of M_*x*_F_*y*_ type, M: metal ion, F: F^–^), with their high fluoride content, show unprecedented NMR detectability of the elements of their inorganic cores while dispersed in solutions based on the NMR-favorable characteristics of ^19^F nuclei (high γ, spin 1/2, and 100% isotopic abundance that results in ∼83% sensitivity compared to ^1^H NMR).^39,40^ Their ^19^F-NMR appearances allowed not only the study of different formation pathways of nanofluorides *in situ*(41) but also their use as imaging agents for *in vivo*^19^F-MRI studies.^39,42,43^ Such robust ^19^F-NMR has the potential to be used for diffusion studies of the core of the colloids. Moreover, uniquely for colloidal nanofluorides, they enable acquisition of NMR signals from both the inorganic core (^19^F-NMR) and the organic ligand (^1^H- or ^31^P NMR) of the same studied colloidal formulation. Therefore, these types of colloidal NCs open new possibilities to explore the diffusion characteristics of dispersed nanoformulations with NMR. Here, we demonstrate that diffusion NMR of the resident elements of the inorganic core of nanofluorides (i.e., ^19^F) are immune to capping ligand dynamics, allowing an accurate determination of the diameters of colloidal NCs dispersed in different media, even in cases of fast ligand exchange. This capability, which was demonstrated for multiple types of nanofluorides, was also used to determine the formation of a protein corona at the surface of inorganic NCs in aqueous solutions, an important feature of their performance in biological systems.

First, four types of nanofluorides, with different core compositions (CaF_2_ or SrF_2_) and different capping ligands, were synthesized to obtain dispersed nanosized colloids in either aqueous solution or in an organic solvent. For the synthesis of the water-dispersed CaF_2_, aminoethyl phosphate (AEP) and citric acid (Cit) were used as capping ligands to obtain AEP-CaF_2_ (Figure 1a, *d*_DLS_ = 4.6 ± 1.1 nm, *d*_TEM[core]_ = 4.0 ± 0.6 nm) and Cit-CaF_2_ (Figure 1b, *d*_DLS_ = 4.9 ± 1.1 nm, *d*_TEM[core]_= 4.5 ± 0.9 nm), respectively. Water-dispersed SrF_2_ NCs were also synthesized with AEP to obtain AEP-SrF_2_, which showed a slightly larger hydrodynamic diameter (Figure 1c, *d*_DLS_ = 7.1 ± 1.7 nm, *d*_TEM[core]_ = 6.9 ± 0.6 nm). Oleic acid was used as a capping ligand for the synthesis of larger and hydrophobic CaF_2_ colloids (OA-CaF_2_, Figure 1d, *d*_DLS_ = 10.0 ± 1.8 nm, *d*_TEM[core]_ = 8.3 ± 1.3 nm) dispersed in organic solvents (i.e., cyclohexane). All types of inorganic nanofluorides were obtained as small-sized (<10 nm) monodispersed colloids in solutions, as reflected by both dynamic light scattering (DLS, Figure 1a–d) and transmission electron microscopy (TEM, Figure S1) studies. The relatively small-size and colloidal nature of all fabricated NCs provide them with characteristic high-resolution ^19^F-NMR spectra with typical peaks resonating at −109 ppm for CaF_2_ (Figure 1e,f,h) and at −88 ppm for SrF_2_ (Figure 1g).^39,41,42^ Note here that the “shoulder” peak, which resonates downfield to the main peak at the ^19^F-NMR spectra, represents the fluorides at the surface of the studied CaF_2_ and SrF_2_ NCs and was assisted before to determine the core size of nanofluorides from their corresponding ^19^F-NMR spectra.^41^

**Figure 1:**
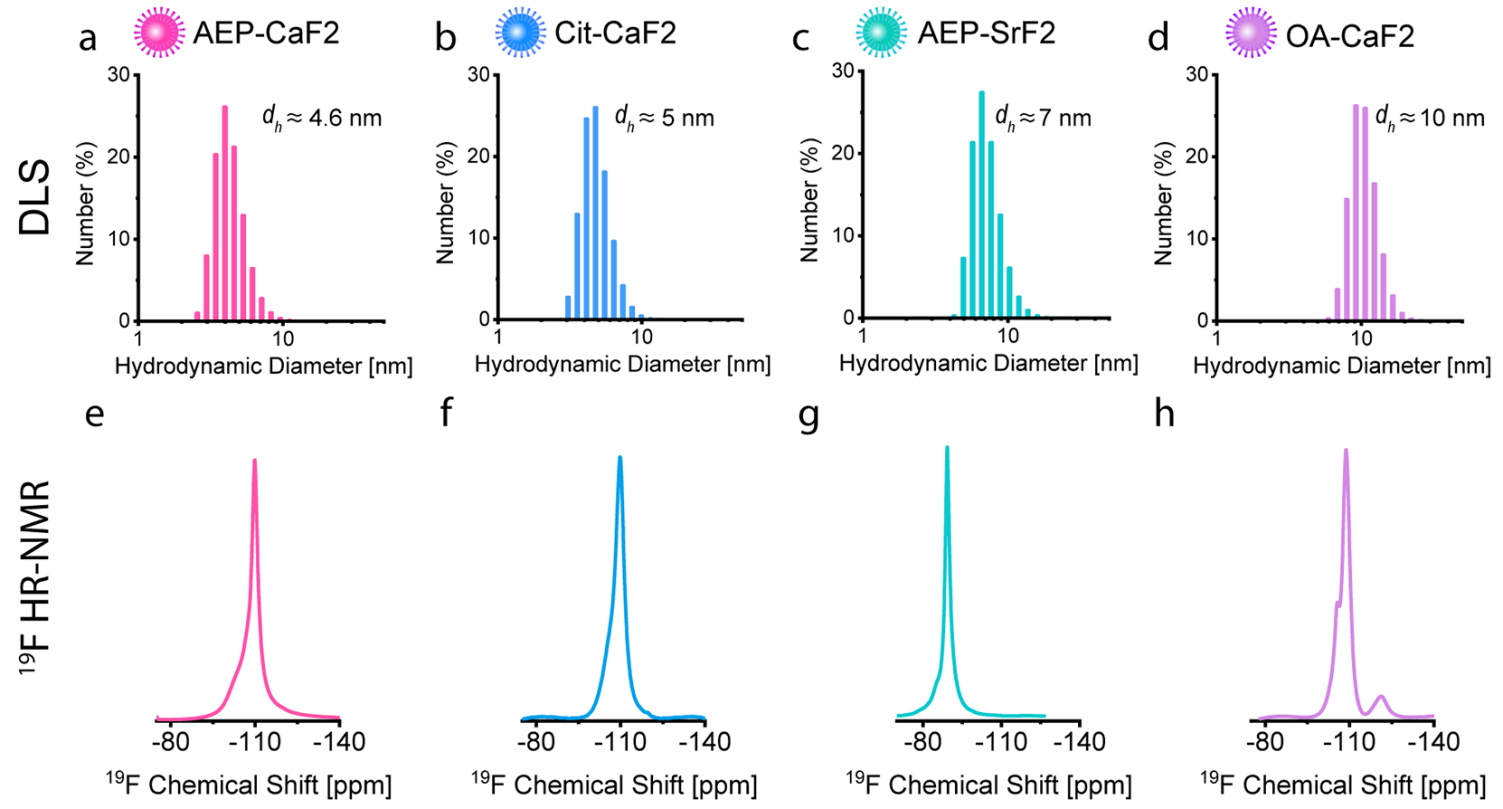
Characterization of nanofluorides. Size distribution of colloidal nanofluorides as measured with DLS for (a) AEP-CaF_2_, *d*_DLS_ = 4.6 ± 1.1 nm, (b) Cit-CaF_2_, *d*_DLS_ = 4.9 ± 1.1 nm, (c) AEP-SrF_2_*d*_DLS_ = 7.1 ± 1.7 nm and for (d) OA-CaF_2_, *d*_DLS_ = 10.0 ± 1.8 nm. High-resolution, liquid-state ^19^F-NMR (376.7 MHz, 298 K) of AEP-CaF_2_ (e), Cit-CaF_2_ (f), AEP-SrF_2_ (g), and OA-CaF_2_ (h). (a–c) and (e–g) Colloids dispersed in D_2_O. (d) and (h) Colloids dispersed in cyclohexane-d12.

This liquid-state ^19^F-NMR detectability of nanofluorides provides a unique platform for the study of their sizes and growth mechanisms at ambient conditions.^41^ Such an NMR feature also has the potential to be used to study the diffusivity of inorganic NCs through the NMR signals of their immobile core elements (i.e., fluorides) independently of the dynamic exchange of their capping organic ligands. Note that the very short T_2_ of the fluorides within these NCs (2.3–5.1 ms, Figure S2) mandates the use of pulsed gradient stimulated echo (PGSTE)-based acquisition schemes rather than pulsed gradient spin echo (PGSE) schemes.^44,45^ This preference also holds for the relatively long T_1_ of the fluorides in the crystal (4–17 s, Figure S2), which allow the use of long diffusion times in PGSTE experiments.^46^ Nevertheless, using a PGSTE sequence for the diffusion ^19^F-NMR experiments was found to be not applicable for diffusion NMR of nanofluorides, likely due to the strong background gradients expected for such solid crystalline materials (Figure S3). To compensate for this effect, we used the bipolar PGSTE (BP-PGSTE) sequence for the diffusion studies,^47−49^ which was found to be essential for diffusion ^19^F-NMR studies of nanofluorides (Figure S3 and supplementary notes).

After optimizing the acquisition parameters required for diffusion ^19^F-NMR studies, a colloidal solution of AEP-CaF_2_ NCs dispersed in D_2_O was first studied (Figure 2). Figure 2a shows, schematically, a studied colloid for which the diffusion NMR could be performed with ^1^H NMR (ligand) or ^19^F-NMR (core), which were not expected to be identical in the case of dynamic ligand exchange. Figure 2b clearly depicts the ^19^F-NMR signal attenuation as a function of the applied diffusion gradient, demonstrating the feasibility and robustness of the proposed diffusion ^19^F-NMR experiments. The normalized ^19^F-NMR signal decay of AEP-CaF_2_ colloids dispersed in aqueous solution as a function of the experimental *b*-values is plotted in Figure 2c (solid spheres). From the slope of the obtained linear signal decay (shown as ln[*I*/*I*_0_]), the diffusion coefficient of AEP-CaF_2_ was evaluated (see Equation S2 and Table S1). Interestingly, when comparing the diffusion characteristics of the colloidal content, i.e., the inorganic core studied with ^19^F-NMR and the organic capping ligand studied with ^1^H NMR, a clear deviation in the diffusion NMR signal decay was obtained (Figure 2c, compare solid spheres with circles). While the diffusion studies performed with ^19^F-NMR represent the relatively slow diffusion that is expected for large colloids, studies performed with ^1^H NMR reveal a much faster diffusion of the AEP capping ligands (Figure 2c and Table S1). This deviation in the diffusion characteristics of the inorganic core and the organic capping ligand could be attributed to the dynamic equilibrium between bound and free ligands and their exchange in solution.^28^

**Figure 2:**
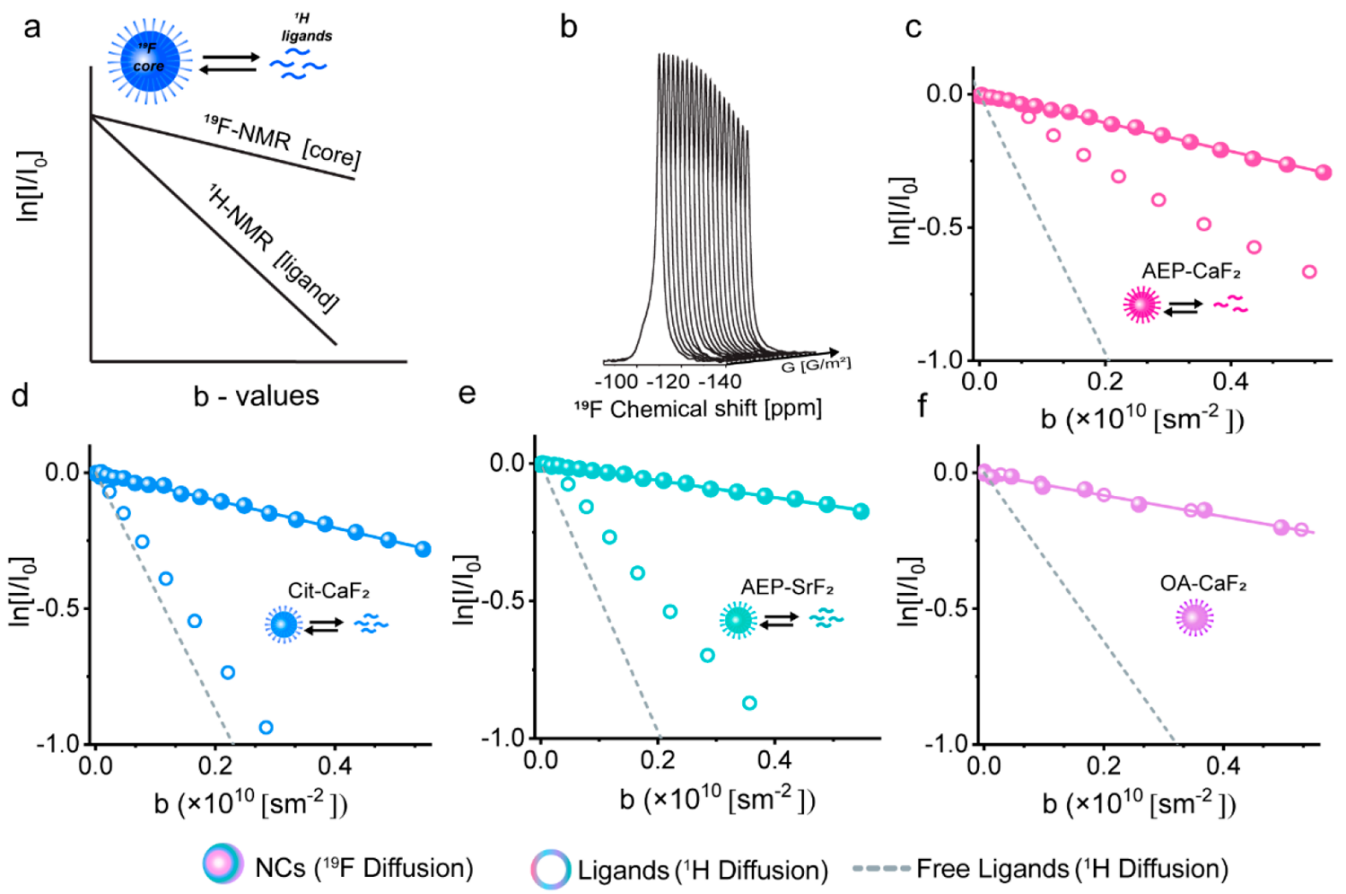
Diffusion experiments of nanofluorides. (a) Schematic illustration of the diffusion NMR characteristics of the ligand (^1^H NMR) and core (^19^F-NMR) content of the studied colloid. (b) Representative stack-plot of ^19^F-NMR signal (of AEP-CaF_2_) decay as a function of the applied diffusion gradient. Logarithmic normalized ^19^F /^1^H signal intensity as a function of the experimental *b*-values obtained from (c) AEP-CaF_2_ in D_2_O, (d) Cit-CaF_2_ in D_2_O, (e) AEP-SrF_2_ in D_2_O, and (f) OA-CaF_2_ in cyclohexane-d12. Colored spheres are the ^19^F diffusion measurements of NCs, colored circles are ^1^H diffusion measurements of ligands in NCs sample, and the dashed gray line is the line fitting of the diffusion NMR of free ligands. In the insets is a schematic of the equilibrium between the ligands and the NCs.

Such dissimilarity of the apparent diffusion properties of the inorganic core (evaluated with ^19^F-NMR) and of the organic capping ligands (evaluated with ^1^H NMR) was observed also for other types of nanofluorides in water, i.e., Cit-CaF_2_ and AEP-SrF_2_ (Figure 2d and Figure 2e, respectively, and Table S1). These observations emphasize, once again, the robustness of ^19^F-NMR diffusion studies of colloidal nanofluorides, which, in contrast to ^1^H NMR studies, are not affected by the dynamic exchange process of the organic ligands between their NC-bound and free state in the studied solution. Note here, that the faster diffusion coefficient obtained from the diffusion ^1^H NMR of exchanging ligands may be compensated to some degree by the use of stronger diffusion weighting (i.e., larger b-values). Such high b-values may, in some cases, result in a biexponential NMR signal decay that could be used to extract the diffusion coefficients of the colloid-bound and free ligand.^50^ For example, for AEP-CaF_2_ colloids dispersed in water, the biexponential fitting of the ^1^H-diffusion NMR signal decay resulted in two diffusion coefficients of two equal populations of AEP (Figure S4). The AEP population that is characterized by slow diffusion characteristics (*D* = 0.72 × 10^-10^ m^2^ s^–1^) shows comparable diffusion coefficient to the one found for the whole colloid from the diffusion ^19^F-NMR experiments (*D* = 0.78 × 10^-10^ m^2^ s^–1^) and therefore may be assigned to bound AEP. For the second population of AEP, a much higher diffusion coefficient was found (*D* = 4.6 × 10^-10^ m^2^ s^–1^) and this population may be assigned to a free AEP diffusing (*D* = 5.2 × 10^-10^ m^2^ s^–1^) in the studied solution. Nevertheless, such an experiment is not applicable to a large excess of free ligand in the studied solutions; it is not valid in the case of very fast exchange dynamics; and it also requires strong gradient systems that are not commonly installed in NMR spectrometers.

In contrast to observations for AEP-CaF_2_ (Figure 2c), Cit-CaF_2_ (Figure 2d), and AEP-SrF_2_ (Figure 2e), when OA-CaF_2_ colloids were studied (Figure 2f), the same diffusion characteristics were obtained from ^1^H NMR and ^19^F-NMR, thus reflecting the negligible exchange process between the CaF_2_-bound and free OA in cyclohexane. This similarity between the diffusion coefficients obtained for the inorganic core (CaF_2_ studied by ^19^F-NMR) and for the organic capping ligand (OA studied by ^1^H NMR) can be attributed, among other factors, to the stronger interactions of the OA ligand with the NCs core or to the strong hydrophobic interactions between adjacent OA ligands.^51,52^

The robustness of the diffusion ^19^F-NMR experiments is further demonstrated in Figure 3a, which shows that nanofluorides with different nanometric sizes can be clearly distinguished one from another by the different signal decay obtained. For the smaller colloids (i.e., AEP-CaF_2_ with *d*_DLS_ = 4.6 ± 1.1 nm and Cit-CaF_2_, *d*_DLS_ = 4.9 ± 1.1 nm), a more pronounce NMR signal decay was obtained compared to a larger formulation that was dispersed in an identical aqueous solution (AEP-SrF_2_ with *d*_DLS_ = 7.1 ± 1.7 nm). Note that the diffusion properties are also dependent on the solvent used and its viscosity and direct comparison of the signal decay obtained for the largest examined colloid (OA-CaF_2_, *d*_DLS_ = 10.0 ± 1.8 nm) to those obtained for nanofluorides in water is meaningless. While the diffusion NMR experiment of OA-CaF_2_ was performed in cyclohexane-d12,^53^ which has a relatively low viscosity (0.9 mPa) the diffusion NMR studies of the other colloids were performed in D_2_O, which has higher viscosity^54^ (1.2 mPa). Therefore, direct comparison of the signal decay without correcting for the solution viscosity is not applicable. Nevertheless, from the slopes of the diffusion NMR signal decays (Figure 2), the diffusion coefficients of all studied formulations could be quantified (using eq S1 and eq S2) when a spherical shape approximation is considered for the studied nanofluorides. From these obtained diffusion coefficient, the colloidal diameter in the studied solution was then evaluated using the Stokes–Einstein relation,^55^eq 1:
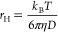
1*r*_H_ is the studied formulation radius (the hydrodynamic diameter of the studied colloid, *d*_H_ = 2*r*_H_), *k*_B_ is the Boltzmann constant, *T* is the temperature at which the experiment was performed, η is the solvent viscosity (1.2 mPa for D_2_O^54^ and 0.9 mPa for cyclohexane-d12^53^), and *D* is the diffusion coefficient. The following diameters were evaluated from the diffusion ^19^F-NMR studies (summarized in Table S1): For AEP-CaF_2_, *d*_diffusion-NMR_ = 4.5 nm (*d*_DLS_ = 4.6); for Cit-CaF_2_, *d*_diffusion-NMR_ = 5.5 nm (*d*_DLS_ = 4.9); for AEP-SrF_2_, *d*_diffusion-NMR_ = 7.7 nm (*d*_DLS_ = 7.1) and for OA- CaF_2_, *d*_diffusion-NMR_ = 9.8 nm (*d*_DLS_ = 10.0). Plotting the diameters of the examined colloids (see Supporting Information for further description), which was found by DLS measurements as a function of the diameters of the same studied colloids found by diffusion ^19^F-NMR experiments, revealed a very good linear correlation (Figure 3b, *R*^2^ = 0.976). Interestingly, for OA-CaF_2_ colloids, for which the stronger interactions of the OA ligand with the NCs core show no exchange of the ligand, one could estimate the length of the ligand by subtracting the core size of the NCs obtained from TEM (*d*_TEM[core]_ = 8.3 nm colloid size from that obtained from the diffusion ^19^F-NMR experiment (*d*_diffusion-NMR_ = 9.8 nm). Such a substantiation results in a 1.5 nm length which is in a good agreement with other reports evaluating the size of OA ligands attached to NCs to be 1.5–2.0 nm.^56^ These results reflect, once again, the robustness, accuracy, and reliability of diffusion ^19^F-NMR for the estimation of the sizes of colloidal NCs *in situ*.

**Figure 3:**
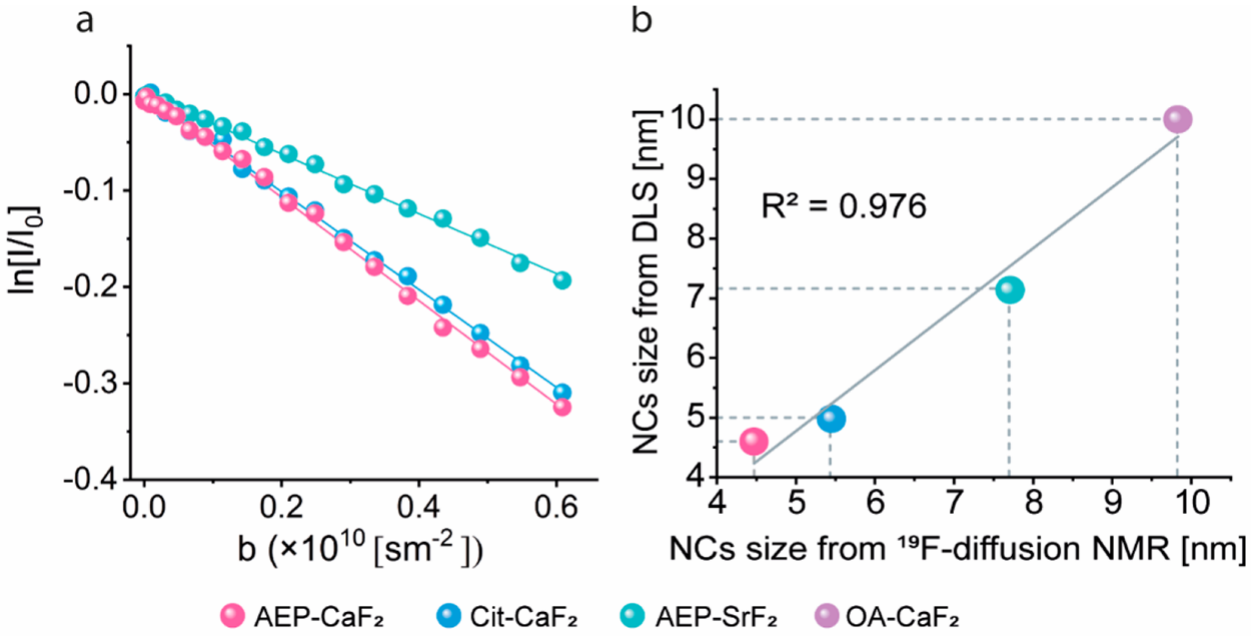
Diffusion ^19^F-NMR of nanofluorides. (a) Logarithmic normalized ^19^F-NMR signal intensity as a function of the experimental *b*-values (eq S1, see supporting notes); AEP-CaF_2_ (pink), Cit-CaF_2_ (blue), and AEP-SrF_2_ (turquoise) dispersed in D_2_O. (b) Correlating NCs diameter evaluated from diffusion ^19^F-NMR and DLS.

Finally, after demonstrating the applicability of the diffusion ^19^F-NMR studies for a range of nanofluorides (Figure 2) and the accuracy of the extracted colloidal diameters (Figure 3), we aimed to examine this approach in the *in situ* formation of large colloidal entities in solutions. To this end, the phenomenon of protein adsorption to the surfaces of NCs, namely the protein corona, which was identified as key for cellular uptake, immune system recognition, biodistribution, and clearance profiles, was studied.^57−59^. This phenomenon is relevant to colloidal nanofluorides as well, as these formulations were extensively developed recently for diverse applications in nanomedicine,^60^ including *in vivo*, ranging from tumor treatment^61,62^ to optical^63−66^ and magnetic resonance imaging.^39,42,43^ Here, we aimed to examine the ability to characterize protein adsorption to nanofluorides using diffusion ^19^F-NMR rather than relying on NMR signals of the organic ligands.^24,25^ Specifically, we studied the effect of introducing proteins (i.e., lysozyme) into aqueous solution of AEP-CaF_2_ on the observed diffusion ^19^F-NMR characteristics of colloidal nanofluorides (Figure 4). An increased amount of protein in the NCs solutions resulted in a clear reduction in the apparent diffusion coefficient of the dispersed nanofluorides, indicating the protein adsorption and the formation of a protein corona at the surface of AEP-CaF_2_. While, for DLS measurements, the light scattering could be attributed to all moieties in solution (e.g., NC-proteins, NCs, and noninteracting proteins), for diffusion ^19^F-NMR studies, the protein content was “invisible” and, thus, provided the ultimate specificity that is required to depict only the effect obtained from proteins that are attached to NCs. This capability may be used in the future to differentiate between a soft (i.e., reversible adsorption) or hard (irreversible adsorption) protein corona,^67^ which mandates quantitative analytical tools for such characterization.

**Figure 4:**
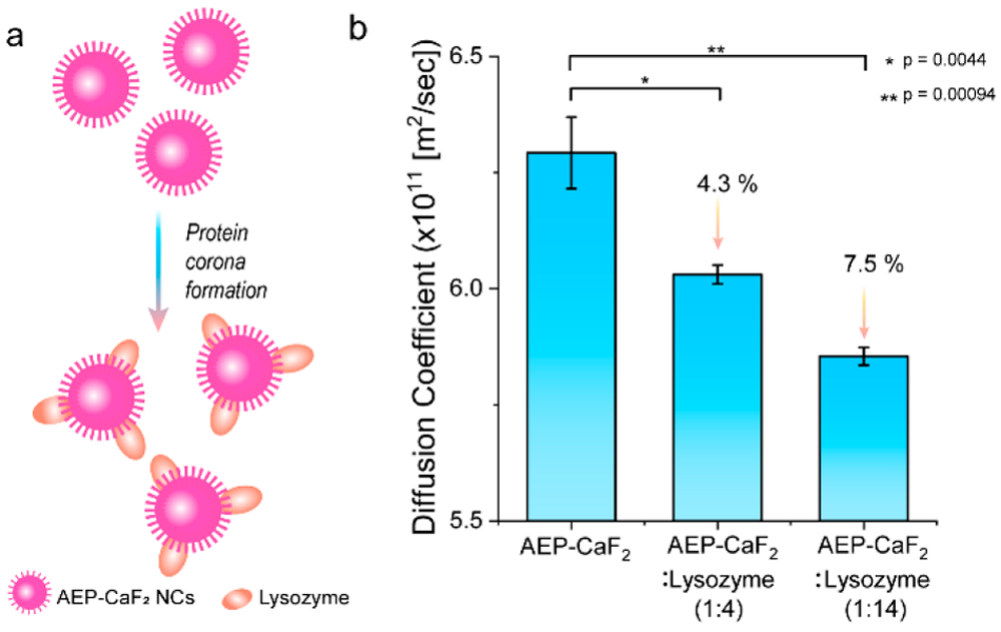
Diffusion ^19^F-NMR of protein adsorption on NCs in water (a) Schematic illustration of the protein adsorption on AEP-CaF_2_ NCs surface. (b) Diffusion ^19^F-NMR of AEP-CaF_2_ in D_2_O before and after the addition of lysozyme proteins with an increased protein-to-nanofluoride ratio, as noted.

To summarize, benefiting from the ability to perform diffusion NMR studies of the organic ligand (^1^H NMR) and of the inorganic core elements (^19^F-NMR), we have studied the translational diffusion characteristics of dispersed colloids. Using a single approach (NMR) to study the diffusion properties of the capping ligands and the solid core of the same colloid, we have demonstrated that, in some cases, the two do not reflect the same diffusion properties. Specifically, we showed that diffusion ^19^F-NMR studies are robust and capable of accurately quantifying the diffusion coefficient of NCs colloids regardless of the dynamic exchange or the excess of surface ligands. Moreover, we have shown that, from these diffusion coefficients, one can determine the diameters of the studied colloids, which were found to be in very good agreement with their sizes found by DLS measurements for a variety of fabrications. Finally, we showed that using diffusion ^19^F-NMR measurements of the inorganic core components of colloidal NCs enables the determination of protein adsorption to their surfaces, and thus, of the formation of a protein corona. Our findings highlight yet another advantage of using nanofluorides as a platform for a better understanding of nanomaterials in solutions.^41,43^ Therefore, it opens an avenue with which to study, *in situ* and at ambient conditions, nanoformulations in solutions with the benefits that NMR-based techniques can provide. Based on NMR principles, the proposed study offers several advantages over other available techniques, among them the resistance to sample turbidity and/or opaqueness that affect light-based measurements. We envision that the strategy described here could be extended to study additional desired properties, such as the diffusion of nanomaterials in different and more complex media, the effect of colloidal charge on the diffusion properties, the determination of the outer shells of nanoformulation, and more. The recent improvements in NMR hardware as well as in ultrafast acquisition schemes should allow the enhanced sensitivity to extend the demonstrated approach to NCs other than nanofluorides. For example, it might be applied to phosphide-, nitride-, or selenide-containing nanomaterials. It might also be applied to study tin-, cadmium-, tellerium-, lead-, or mercury-based NCs, but these types of materials may still suffer from low sensitivity when studied with liquid-state NMR.
