# Supplementary material for: Diffusion 19F-NMR of Nanofluorides: In Situ Quantification of Colloidal Diameters and Protein Corona Formation in Solution
Source: Nano Lett. 2022 Oct 18;22(21):8519–25. doi: 10.1021/acs.nanolett.2c02994 (PMC9650773; doi:10.1021/acs.nanolett.2c02994)
Supplement: Supplementary file 1 — nl2c02994_si_001.pdf [file nl2c02994_si_001.pdf]

# Supporting Information

## Diffusion $^{19}$F-NMR of nanofluorides: In situ quantification of colloidal diameters and protein corona formation in solution

Reut Mashiach[1], Liat Avram[2] and Amnon Bar-Shir[1*]

[1]Department of Molecular Chemistry and Material Science, [2]Department of Chemical Research Support, Rehovot, 7610001, Israel

*Corresponding Author: amnon.barshir@weizmann.ac.il

# Contents

Methods .......... 3
NMR measurements .......... 4
NCs Synthesis .......... 5
Supporting Figures .......... 7
Supplementary notes .......... 9
Supplementary Equations .......... 11
Supporting Tables .......... 13
References .......... 14

# Methods

## NMR

NMR experiments were performed on a 9.4 T AVANCE III NMR spectrometer (Bruker, Germany), at 298 K. Prior to the $^{19}$F-NMR experiments (376.7 MHz), $^{1}$H-NMR spectra (400.35 MHz) were acquired for all samples.

$^{19}$F and $^{1}$H NMR diffusion study of protein corona formation were performed on an 11.74T AVANCE III HD NMR spectrometer (Bruker, Germany), at 298 K.

## Transmission Electron Microscopy (TEM)

TEM and high-resolution TEM (HRTEM) images of all NCs samples were acquired with a JEOL JEM 2100 high-resolution electron microscope. With Gatan Digital Micrograph and an accelerating voltage of 200 kV with a beam source of an LaB6 thermal emission, and a bottom-mount CCD camera (Gatan Ultra scanXP 2k x 2k).

## Reactor

Solvothermal reaction of OA-$CaF_2$ was synthesized in a compact micro reactor (Parr Instruments Company, model 5500). The reactor has. The reaction takes place inside a 100ml Teflon liner with an internal thermocouple to maintain 160°C in addition to the internal stirrer with a continuous stirring.

## Dynamic Light Scattering (DLS)

The hydrodynamic diameter and size distribution of the obtained nanoparticles were evaluated by dynamic light scattering, Malvern Nano-ZS. Measurements were taken in a 12.5 mm diameter cylindrical quartz cuvette (for cyclohexane dispersed NCs) or plastic (for water dispersed NCs) according to the appropriate solvent viscosity.

## NMR measurements

Number of scans for each $^1H$ NMR (NS) NS= 20, $^{19}F$ spectra were acquired with NS=64. $T_1$ and $T_2$ of NF: $T_1$ relaxation was measured with the inversion recovery sequence and $T_2$ relaxation was calculated using the Car-Purcell-Meiboom-Gill (CPMG) experiment (Fig. S2).

<u>Diffusion NMR</u>

NMR diffusion measurements were performed with a magnetic field pulse gradients in the z-direction of $G_{max} = 50\ Gauss/cm$. Both $^1H$ and $^{19}F$ NMR diffusion measurements were performed with BPSTE. For $^1H$ ligands diffusion measurements of we used ($\gamma/_{2\pi} = 42.58 MHzT^{-1}$)[1], $\delta = 1msec$ and $\Delta = 100msec$ for AEP-$SrF_2$ and Cit-$CaF_2$ , and $\delta = 6msec$ and $\Delta = 60msec$ for OA-$CaF_2$.

For $^{19}F$ diffusion measurements ($\gamma/_{2\pi} = 40.04 MHzT^{-1}$)[1], $\delta = 1.2msec$ and $\Delta = 600msec$ for AEP-$CaF_2$, AEP-$SrF_2$, Cit-$CaF_2$ and $\delta = 1.2msec$ and $\Delta = 800msec$ for OA-$CaF_2$. Linear line fitting was performed on the plot of $ln(I/I_0)$ versus $b = \gamma^2\ \delta^2\ G^2\ (\Delta - \delta/3)$ and the slope measured is the diffusion coefficient (Fig.3 Eq.S1. and Eq.S2.).

<u>Diffusion NMR for protein corona formation measurements</u>

NMR diffusion measurements (before and after addition of Lysozyme proteins) were performed with a magnetic field pulse gradients in the z-direction of $G_{max} = 50\ Gauss/cm$. Both $^1H$ and $^{19}F$ NMR diffusion measurements were performed with BPSTE sequence. $^1H$ solvent diffusion measurements for calibration was measured with ($\gamma/_{2\pi} = 42.58 MHzT^{-1}$)[1], $\delta = 1msec$ and $\Delta = 100msec$. For $^{19}F$ NCs diffusion measurements we used ($\gamma/_{2\pi} = 40.04 MHzT^{-1}$)[1], $\delta = 1.2msec$ and $\Delta = 600msec$, number of gradient increment was 20. Each $^1H$ and $^{19}F$ diffusion measurement was performed 3 times consecutively and an average was calculated and reported. The diffusion constants presented in Figure 5 include the calibration of the solvents (according to equation S3).

# NCs Synthesis

AEP-$MF_2$ (M= $Ca^{2+}$ or $Sr^{2+}$)

A solution of (0.17M) AEP and NaF (0.08M) in 25ml of dd$H_2O$ at pH=7, neutralized with ammonium hydroxide with was heated to 60°C while stirring. Then, 1ml of $Ca(NO_3)_2$ (0.04M) of $SrCl_2$ (0.04M) was rapidly injected to the hot solution and left for 100min at 60°C. Then the solution was removed from the heating plate and cooled to room temperature. For purifications the solution was centrifuged and washed with ethanol/dd$H_2O$. Additional purification procedure was achieved by dialysis vs. water in order to remove traces of unreacted reagents and free ligands.

OA-$CaF_2$

4.2 ml of oleic acid was mixed together with 12 ml of ethanol and 0.1 gr of sodium hydroxide until a homogeneous milky mixture evolved. Then, 2mmol of $Ca(NO_3)_2$ in 5 ml of water were added to the solution followed by an immediate addition of aqueous solution of 4mmol of NaF in 5 ml of water, under vigorous stirring in a 50 ml flask. The solution was stirred for 1h and transferred to a 100ml Teflon liner placed inside the reactor. After 16h at 160°C and continuous gentle stirring, the system was cooled to room temperature and the solution was centrifuged at 8500rpm for 10 min to obtain the powder products and washed in ethanol/ cyclohexane two times to remove impurities. The obtained NCs were dispersed in an organic solvent of cyclohexane.

Cit-$CaF_2$

1mmol of NaF was added to 820mg citric acid in 50ml of *dd*$H_2O$ at pH=7, and neutralized with ammonium hydroxide. The solution was heated to 75°C while stirring. Then, 1/2mmol $CaCl_2$ was dissolved in 2ml of *dd*$H_2O$ and was rapidly injected to the hot solution. The solution was removed from the heating plate immediately after the injection and cooled to room temperature. Then, the solution was centrifuged and washed with ethanol/*dd*$H_2O$.

## NMR Sample preparation

For aqueous dispersed NCs of AEP-$MF_2$ (M= $Ca^{2+}$ or $Sr^{2+}$) or Cit-$CaF_2$; the obtained NC solution was dried for 20min under $N_2$ to remove the solvents and replaced with deuterated solvent of $D_2O$.

For organic solvent dispersed NCs of OA-$CaF_2$; the obtained NC solution was vacuum-dried for 40min to remove the solvents and replaced with deuterated solvent of d-12 Cyclohexane.

## NMR sample preparations for protein corona formation on AEP-$CaF_2$ NCs

For quantifying the number of NCs in a 400 µl NMR sample we first acquired a $^{19}F$ spectrum of the AEP-$CaF_2$ solution with a capillary filled with trifluoroacetate (with a known $^{19}F$ atoms concentration), we found that the concentration is 765mM [$^{19}F$ atoms] .

The number of $^{19}F$ atoms in 400 µl solution is:

$$400 \times 10^{-6} liter \times 765 \times 10^{-3} \frac{mol}{liter} \times 6.023 \times 10^{23} \frac{atoms}{mol} = 1.84 \times 10^{20} \quad atoms$$

Using Equation S4 (number of $^{19}F$ atoms per $CaF_2$ NC taking r=2 as calculated from the TEM image in Fig.S1a) we could calculate the number of NCs in a 400 µl sample:

$$N_{NCs} = \frac{1.84 \times 10^{20} \quad atoms}{204 \times 2^3 \ atoms/_{NC}} = 1.12 \times 10^{17} \ NCs$$

For a ratio of 1:4 (NCs : Lysozyme proteins):

40µl from a stock solution of 18.6 $mM$ of Lysozyme powder in $D_2O$ (Lysozyme from chicken egg white purchased from Sigma Aldrich) was mixed with 400 µl sample of AEP-$CaF_2$ and pipetted gently. After 1h NMR measurements were conducted.

For a ratio of 1:14 (NCs : Lysozyme proteins):

40µl from a stock solution of 66.33 $mM$ of Lysozyme powder in $D_2O$ (Lysozyme from chicken egg white purchased from Sigma Aldrich) was mixed with 400 µl sample of AEP-$CaF_2$ and pipetted gently. After 1h NMR measurements were conducted.

For control of AEP-$CaF_2$ (no proteins):

40µl of $D_2O$ was added to 400 µl of AEP-$CaF_2$ NCs solution.

# Supporting Figures

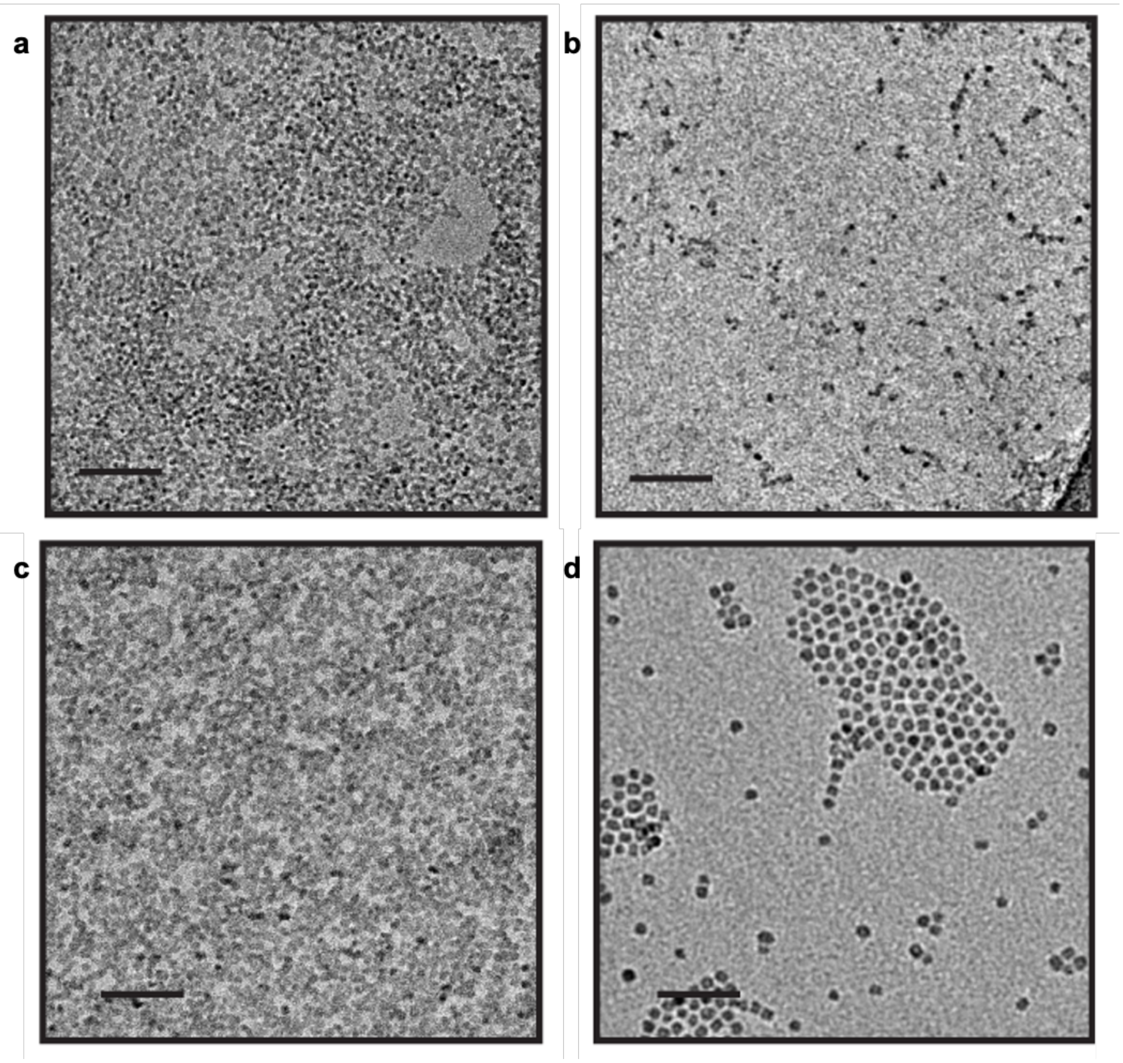


**Figure S1. TEM images of the fabricated Nanofluorides** (a) AEP-$CaF_2$. (b) Cit-$CaF_2$. (c) AEP-$SrF_2$. (d) OA- $CaF_2$. Scale bar is 50 nm. The calculated diameters of the cores of the synthesized NCs were found to be: (a) AEP-$CaF_2$: $d_{TEM[core]}$=4.0±0.6nm, (b) Cit-$CaF_2$: $d_{TEM[core]}$=4.5±0.9nm, (c) AEP-$SrF_2$: $d_{TEM[core]}$=6.9 ±0.6nm, (d) OA-$CaF_2$: $d_{TEM[core]}$=8.3±1.3nm.

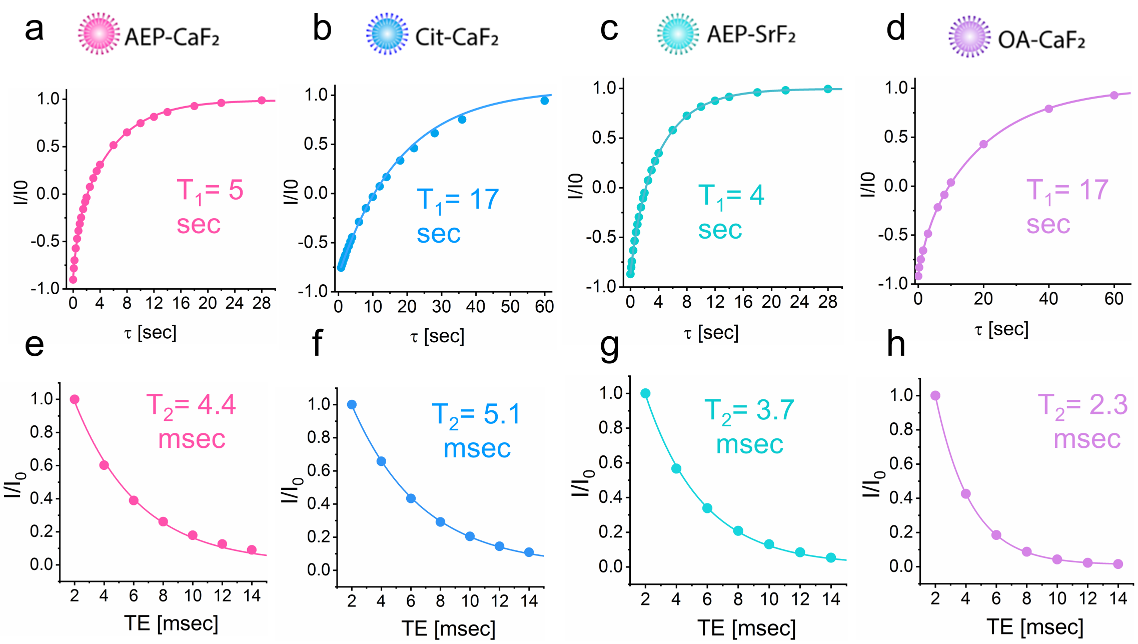


**Figure S2. $^{19}$F NMR relaxation values of the various nanofluoride in solution.** $^{19}$F-signal of NCs as a function of inversion time obtained from $^{19}$F-NMR inversion-recovery experiments of (a) AEP-$CaF_2$ in $D_2O$. (b) Cit-$CaF_2$ in $D_2O$. (c) AEP-$SrF_2$ in $D_2O$. (d) OA- $CaF_2$ in cyclohexane-$d_{12}$ and the calculated $T_1$ values. The normalized $^{19}$F signal as a function of the echo time in CPMG experiments and the calculated $T_2$ relaxation values of the various nanofluorides in solution. (e) AEP-$CaF_2$ in $D_2O$. (f) Cit-$CaF_2$ in $D_2O$. (g) AEP-$SrF_2$ in $D_2O$ (h) OA- $CaF_2$ in cyclohexane-$d_{12}$.

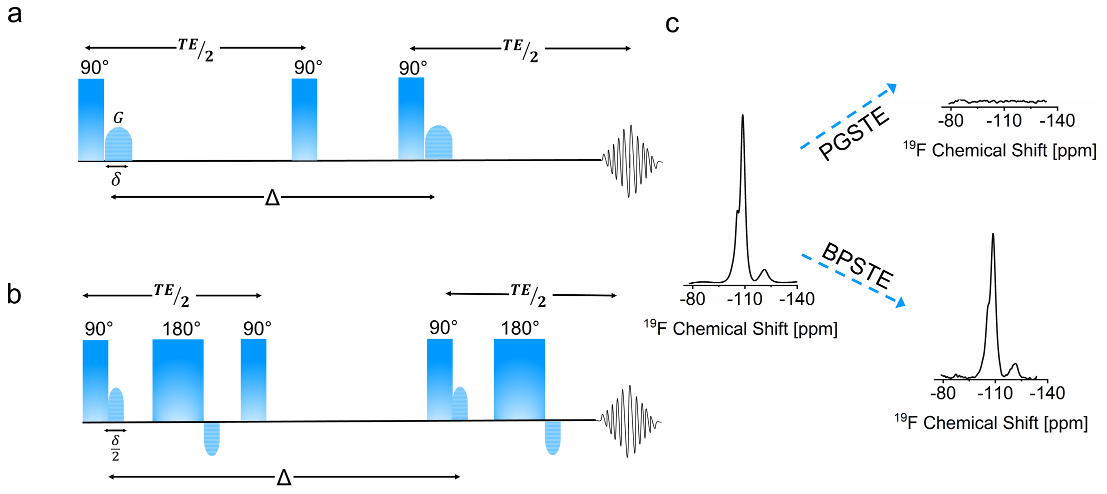


**Figure S3. $^{19}$F-Diffusion NMR of nanofluorides.** (a) Schematics of the Pulsed Gradient Stimulated Echo (PGSTE) pulse sequence. (b) Schematics of the Bi-polar Stimulated Echo (BP-STE) pulse sequence. $G$ represents the amplitude of the applied magnetic field gradient, $\delta$ the magnetic field gradient pulse duration, $\Delta$ the diffusion time, and TE is the echo time. (c) $^{19}$F-NMR signal of OA-$CaF_2$ NCs dispersed in cyclohexane-$d_{12}$ without applying the diffusion gradients and after applying G= 0.681 Gauss/cm with either the PGSTE sequence (top spectrum) or the BP-STE sequence (bottom spectrum).

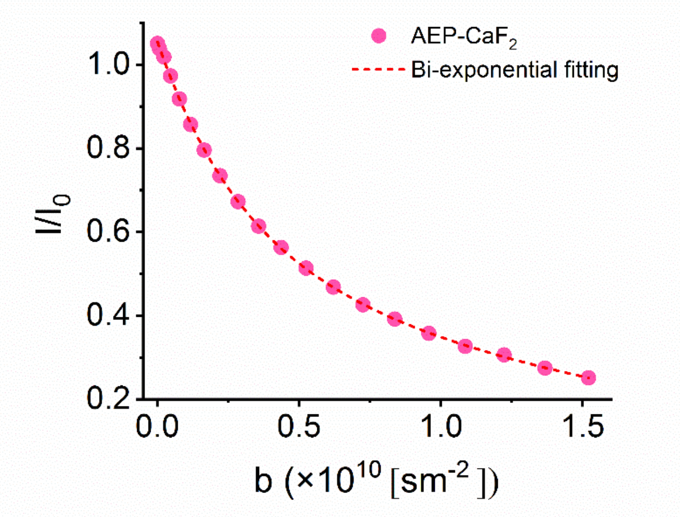


**Figure S4. Bi-exponential fitting of the $^1$H-diffusion NMR experiment of AEP-$CaF_2$ colloids dispersed in water.** the studied colloid. Colored spheres represent the diffusion NMR signal decay obtained from the $^1$H-NMR peaks of the AEP ligand. Dashed line represents the bi-exponential fitting of the experimental data resulted in two equal populations of AEP, one that is represented by a slow diffusion coefficient (D= $0.72\times10^{10}$ $m^2$ $sec^{-1}$) and one by a fast diffusion coefficient (D= $4.6\times10^{10}$ $m^2$ $sec^{-1}$). For comparison, the diffusion coefficient of the free AEP ligand in water was found to be D= $5.2\times10^{10}$ $m^2$ $sec^{-1}$ and the diffusion coefficient of the AEP-$CaF_2$ colloids as extracted from $^{19}$F-NMR experiment was evaluated to be D= $0.78\times10^{10}$ $m^2$ $sec^{-1}$.

# Supplementary notes

## Sequence optimization for $^{19}F$ diffusion of NCs

To examine the feasibility of performing $^{19}F$-diffusion NMR experiments, a solution containing OA-$CaF_2$ in cyclohexane was first studied using a PGSTE-sequence. Interestingly, even when a very weak pulse gradient was applied (G= 0.681 gauss/cm) for a duration of only 1.2 msec in the $^{19}F$-NMR PGSTE experiment, the characteristic high-resolution $^{19}F$-NMR signal of the studied OA-$CaF_2$ was totally eliminated (Fig. S3, top spectrum). This signal elimination might be a result of local magnetic susceptibilities that are expected in solid crystalline materials such as that of the nanofluoride core. In a static magnetic field this may result in background gradients ($G_0$), which could lead to unwanted NMR signal attenuation. Moreover, significant cross-terms between $G_0$ and the applied diffusion gradients may introduce systematic errors to the calculated diffusion coefficients[2]. To reduce any effect of $G_0$ on the obtained diffusion measurements, an alternative to the PGSTE-sequence is frequently used in which the gradient pulses (G) are replaced by two gradients with a half duration ($\delta/2$) and opposite phases that are separated by a 180° rf pulse and called bi-polar gradients (BP-STE, Fig. S3b). Indeed, when a BP-STE sequence was used to acquire diffusion $^{19}F$-NMR data, a clear and characteristic spectrum was detected even upon the application of diffusion gradients (Fig. S3c, bottom spectrum)[3]. This observation of the canceling out of $G_0$ effects when using BP-STE is in good correlation with previous reports that showed the same phenomenon in other samples with increased magnetic susceptibilities, such as liquid crystals[4], porous materials[5], and minute compartments[6].

# Supplementary Equations

## Equation S1

The normalized $^{19}$F-NMR signal decay of the nanofluorides as a function of the experimental b-values is plotted in Fig. 2c-f and were calculated according to Equation S1:

$$b = \gamma^2 G^2 \delta^2 (\Delta - \frac{\delta}{3}) \quad \text{(Eq. S1)}$$

where $\gamma$ is the gyromagnetic ratio of the studied nuclei ($^{\gamma}/_{2\pi} = 40.04\ MHzT^{-1}$for $^{19}$F)[1], $G$ is the applied gradient, $\delta$ is the duration time of the applied $G,$ and $\Delta$ is the diffusion time.

## Equation S2

Importantly, using Equation 2, the diffusion coefficient, D, can be extracted from the linear slope of the plot in Fig. 2c-f or Fig.3a:

$$\ln \frac{I}{I_0} = -b \cdot D \quad \text{(Eq. S2)}$$

where $I$ is the $^{19}$F-NMR signal at a given gradient strength (G) and $I_0$ is the signal without the pulsed-gradient application; D is the apparent diffusion coefficient.

## Equation S3

In order to consider both the viscosity of the NCs solution (compared to the pure solvent viscosity η) and the gradient calibration constants[7] we calculated a calibration factor ($C_f$) in Eq S3;

$$C_f = \frac{D_{solvent\ known}}{D_{solvent\ measured}} \quad \text{(Eq. S3)}$$

Where $D_{solvent\ measured}$ is the diffusion coefficient measured from the diffusion NMR experiment of the solvent in the studied solution and $D_{solvent\ known}$ is the diffusion coefficients from literature (values in Table S2).

## Equation S4

In order to calculate the number of NCs in the NMR tube (400 µl of solution) we first estimate the number of $^{19}$F atoms per $CaF_2$ NC;

$Moles\ of\ atoms\ per\ CaF_2\ NC\ is$

$$nCaF_2 = \frac{d_{CaF_2} \times V_{CaF_2}}{M_{w_{CaF_2}}}$$

$Where\ the\ density\ is{:}\ d_{CaF_2} = 3.18\ {}^{gr}/_{cm^3}$ ,

$The\ molecular\ weitgh\ is{:}\ M_{w_{CaF_2}} = 78.07\ {}^{gr}/_{mol}$

$And\ the\ volume\ of\ CaF_2\ (with\ radius\ r)\ is:\ V_{MF_2} = \frac{4\pi\ (r)^3}{3}$

$The\ number\ of\ {}^{19}F\ atoms\ per\ CaF_2\ NC\ (knowing\ that\ for\ each\ CaF_2\ there\ are\ two\ {}^{19}F\ atoms){:}$

$2 \times nCaF_2 \times N_A$

$Number\ of\ {}^{19}F\ atoms\ per\ CaF_2 = 204.7 \times r^3$ (Eq. S4)

# Supporting Tables

**Table S1**. Calculated diffusion constant of different nanofluorides as evaluated from $^{19}F$- diffusion NMR experiments. Also shown the calculated radii of the nanofluorides obtained from Stokes-Einstein relation, Eq. 1.

| Sample | Apparent diffusion constants of **ligands** $^{1}H$ diffusion ($\times 10^{10}$ [$\frac{m^2}{sec}$]) | Calibrated diffusion constants of **ligands** $^{1}H$ diffusion ($\times 10^{10}$ [$\frac{m^2}{sec}$]) | Apparent diffusion constants of **NCs** $^{19}F$ diffusion ($\times 10^{10}$ [$\frac{m^2}{sec}$]) | Calibrated diffusion constants of **NCs** $^{19}F$ diffusion ($\times 10^{10}$ [$\frac{m^2}{sec}$]) | Calculated radius of **NCs** [$nm$] | Calculated diameter of **NCs** [$nm$] |
|---|---|---|---|---|---|---|
| AEP-$CaF_2$ | 1.81 | 2.57 | 0.54 | 0.78 | 2.24 | 4.48 |
| Cit-$CaF_2$ | 3.32 | 4.04 | 0.51 | 0.64 | 2.73 | 5.46 |
| AEP-$SrF_2$ | 2.92 | 4.11 | 0.31 | 0.45 | 3.86 | 7.72 |
| OA-$CaF_2$ | 0.40 | 0.51 | 0.39 | 0.49 | 4.92 | 9.84 |

**Table S2**. Empirical diffusion constants of the solvents in NCs solutions and diffusion constants from literature (for $D_2O$[8] and Cyclohexane-$d_{12}$[9]).

| Sample | Empirical diffusion constants for solvents ($\times 10^{9}$ [$\frac{m^2}{sec}$]) | Diffusion constants for solvents ($\times 10^{9}$ [$\frac{m^2}{sec}$]) from literature |
|---|---|---|
| AEP-$CaF_2$ | 1.34 | 1.9[8] |
| Cit-$CaF_2$ | 1.56 | 1.9[8] |
| AEP-$SrF_2$ | 1.35 | 1.9[8] |
| OA-$CaF_2$ | 1.1 | 1.39[9] |

# References


1. Lide, D. R., *CRC handbook of chemistry and physics*. CRC press: 2004; Vol. 85.
2. Stejskal, E. O.; Tanner, J. E., Spin Diffusion Measurements: Spin Echoes in the Presence of a Time-Dependent Field Gradient. **1965,** *42* (1), 288-292.
3. Cotts, R.; Hoch, M.; Sun, T.; Markert, J. J. J. o. M. R., Pulsed field gradient stimulated echo methods for improved NMR diffusion measurements in heterogeneous systems. J. Magn. Reson. **1989,** *83* (2), 252-266.
4. Furó, I.; Dvinskikh, S. V., Field Gradient NMR of Liquid Crystals. In *Modern Magnetic Resonance*, Webb, G. A., Ed. Springer Netherlands: Dordrecht, 2006; pp 117-122.
5. Callaghan, P.; MacGowan, D.; Packer, K. J.; Zelaya, F. O., Influence of field gradient strength in NMR studies of diffusion in porous media. *Magn. Reson. Imaging.* **1991,** *9* (5), 663-671.
6. Bar-Shir, A.; Avram, L.; Ozarslan, E.; Basser, P. J.; Cohen, Y., The effect of the diffusion time and pulse gradient duration ratio on the diffraction pattern and the structural information estimated from q-space diffusion MR: experiments and simulations. *J. Magn. Reson.* **2008,** *194* (2), 230-6.
7. Gibbs, S. J.; Johnson, C. S., A PFG NMR experiment for accurate diffusion and flow studies in the presence of eddy currents. *J. Magn. Reson.* **1991,** *93* (2), 395-402.
8. Claridge, T. D., *High-resolution NMR techniques in organic chemistry*. Elsevier: 2016; Vol. 27.
